# A Genotype of Modified Vaccinia Ankara (MVA) that Facilitates Replication in Suspension Cultures in Chemically Defined Medium

**DOI:** 10.3390/v5010321

**Published:** 2013-01-21

**Authors:** Ingo Jordan, Deborah Horn, Katrin John, Volker Sandig

**Affiliations:** ProBioGen AG, Goethestr. 54, 13086 Berlin, Germany; E-Mails: deborah.horn@probiogen.de (D.H.); katrin.john@probiogen.de (K.J.); volker.sandig@probiogen.de (V.S.)

**Keywords:** modified vaccinia Ankara, MVA, vaccine, duck suspension cell line, AGE1.CR.pIX

## Abstract

While vectored vaccines, based on hyperattenuated viruses, may lead to new treatment options against infectious diseases and certain cancers, they are also complex products and sometimes difficult to provide in sufficient amount and purity. To facilitate vaccine programs utilizing host-restricted poxviruses, we established avian suspension cell lines (CR and CR.pIX) and developed a robust, chemically defined, culturing process for production of this class of vectors. For one prominent member, modified vaccinia Ankara (MVA), we now describe a new strain that appears to replicate to greater yields of infectious units, especially in the cell-free supernatant of cultures in chemically defined media. The new strain was obtained by repeated passaging in CR suspension cultures and, consistent with reports on the exceptional genetic stability of MVA, sequencing of 135 kb of the viral genomic DNA revealed that only three structural proteins (A3L, A9L and A34R) each carry a single amino acid exchange (H639Y, K75E and D86Y, respectively). Host restriction in a plaque-purified isolate of the new genotype appears to be maintained in cell culture. Processing towards an injectable vaccine preparation may be simplified with this strain as a complete lysate, containing the main burden of host cell contaminants, may not be required anymore to obtain adequate yields.

## 1. Introduction

Conventional killed vaccines predominantly elicit antibody responses and, for this reason, may not be sufficient in the fight against some chronic infectious diseases. Live vaccines appear to be better suited to these challenges as they usually induce a broad immune response that also involves the cellular compartment. However, with increasing numbers of immunocompromized individuals, and the expansion of international mobility, the use of replication-competent strains can be associated with unacceptable risks [1,2,3]. Modern vectored vaccines combine the advantages of an attenuated infection with the safety of host-restricted vectors. Especially promising vectors are hyperattenuated poxviruses, including modified vaccinia Ankara (MVA). MVA has been attenuated by repeated passaging on chicken-derived material as production substrate [4]. In the course of this attenuation, approximately 15% of the genomic DNA at multiple sites was lost [5]. Compared to parental vaccinia virus, MVA has a very narrow host range and replication in human cells is blocked or severely impaired [6,7,8].

MVA has demonstrated safety in numerous clinical trials [9,10,11,12,13] and is an efficient stimulator of the immune response [14,15,16,17]. However, to provide an adequate supply of hyperattenuated vectors can be challenging: they have to be given at high doses [18] because they replicate to very low levels, or not at all in the recipient, and they require special host cells for production. Currently, vaccines adapted to avian substrates are being produced in material obtained from embryonated chicken eggs [19]. However, dependence on primary animal-derived material that has to be continuously fed into vaccine production processes is not an optimal situation. To alleviate this problem we have designed and generated two related, fully permissive, avian cell lines, CR and CR.pIX [20], and based on these cell lines, further developed a robust, chemically defined, and highly efficient production process for hyperattenuated vectors [21,22]. Cell banks of CR.pIX have been exhaustively tested according to regulatory guidelines against adventitious agents, including circoviruses and retroviruses. The cell line is derived from a single Muscovy duck embryo, and proliferates in true suspension without microcarriers. All manipulation steps are extensively documented, from isolation and immortalization of primary cells, to adaptation to chemically defined medium.

Although the avian CR cell line is fully permissive for MVA, we studied replication of serial virus passages in the chemically defined environment and were surprised to notice a further increase in titers within 10 such passages. Investigation of isolates at various points of the passaging revealed accumulation of a distinct genotype, which we believe confers an advantage to replication in suspension cultures. In adherent cultures this genotype appears to have only a limited advantage over the wildtype and is not enriched. The new virus lineage and the associated virus properties are further described here.

## 2. Results

### 2.1. Passaging of MVA in Suspension Cultures

**Figure 1:**
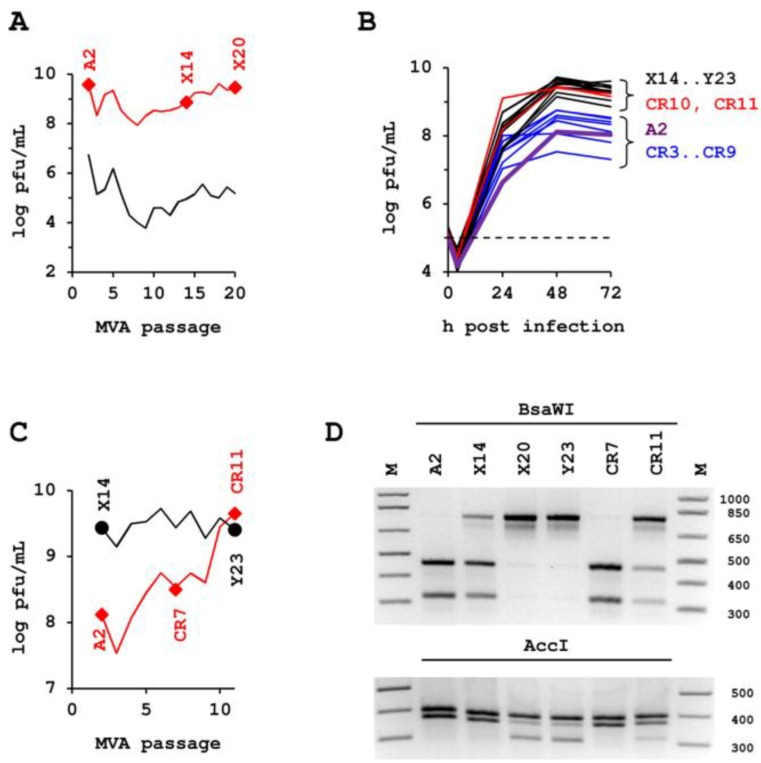
Effect of successive passages in CR suspension culture in chemically defined media and via induction of aggregates on yield and genotype of modified vaccinia Ankara (MVA). (**A**) to (**D**) The various lineages are marked with letters and the respective passage number is attached to the letter: MVA-A2 is the seed preparation, MVA-X3 to MVA-X20 describes the lineage of first passaging experiment, MVA-CR3 to MVA-CR19 the lineage of the second passaging experiment. (**A**) Passage of MVA with variable amount of input virus. The concentration of infectious units in the complete lysate used to inoculate the culture is shown in the lower curve (black line), the resulting yield in the upper curve (red line). Cell concentration was adjusted to 2 × 10^6^ cells/mL at the time of infection for all passages. Yield of infectious units (usually 72 h post infection) was estimated from extent of CPE in the suspension culture. With this estimate infection for the next passage was performed. Actual MOI was determined retrospectively after titration of several passages in parallel and averages for the whole series at 0.096. (**B**) Serial passaging of MVA with precise MOI of 0.05 for each iteration. Shown is the superimposition of all time kinetics of high (>14) and low (starting with two) passages of MVA and concentration of input virus (stippled line). (**C**) Peak titers in the complete lysate in (**B**) usually were obtained 48 h post infection and are shown in this chart for the two parallel experiments for each virus passage. The passaging series that was started with the seed virus preparation MVA-A2 is shown in red, the reference passaging series that was started with the high passage isolate MVA-X14 from the experiment shown in (**A**) is depicted in black. (**D**) Accumulation of the D86Y genotype confirmed by restriction fragment length polymorphism.

Scalable and efficient production processes are required to supply global vaccine programs with sufficient infectious units of hyperattenuated vectors. Scalability can be best achieved with suspension cultures and robust production is facilitated by using chemically defined media. However, virus replication in a cell suspension not supported by microcarriers and in a medium characterized by negligible protein content, and composition free of serum and hydrolysates, is very distant to the natural environment. To increase MVA titers we induce cell aggregates to facilitate cell-to-cell spread and harvest the infected host cell in addition to culture supernatant [21]. Although the CR and CR.pIX cell lines are fully permissive for MVA, and the aggregate-based production process results in very high titers beyond 10^8^ pfu/mL, yields for MVA passaged in this environment appears to increase further (Figure 1). The starting generation was ATCC #VR-1508 MVA, amplified on primary chicken embryo fibroblasts once, followed by one passage on the adherent CR cell line in the presence of serum. This seed virus, separated by two passages from the ATCC preparation, is called MVA-A2 in our study. In a first passaging experiment, virus yield from each preceding passage was estimated and, with this estimate, we aimed to infect the subsequent culture with an MOI of 0.01 to 0.1. Because of the time required until titrations can be evaluated, the true MOI varied from passage to passage and in our case (any passaging could also have an attenuating or dampening effect on virus replication) yields and MOI appeared to increase with increasing passage. After 20 passages in the chemically-defined suspension, process amplification (the ratio of yield to input virus) of the resulting generation was beyond 15000-fold, as opposed to 3000-fold in the initial infections (Figure 1A and Figure 6B).

MVA is known for its exceptional genetic stability [23,24]. To confirm any passaging effects, the experiment was repeated such that parallel CR suspension cultures were infected in duplicate with an intermediate isolate of the first passaging experiment (called MVA-X14) and again with MVA-A2. Each subsequent passage was performed with the 48 h-harvest of one (arbitrarily chosen) of the two independent threads of the previous passage. As opposed to the initial blind passaging, the second experiment was performed with virus titered prior to infection so that a consistent MOI of 0.05 could be adjusted for each passage. The virus lineage obtained from this passaging experiment, also starting from isolate MVA-A2, is called MVA-CR; the virus lineage that extends previous isolate MVA-X14 was called MVA-Y.

Again we observed gradually increasing titers, and for this reason isolated and sequenced the genomic DNA of MVA-A2, MVA-CR7, and MVA-CR11. Assembly of the contigs was performed with MVA GenBank entry #U94848 as guide sequence and in an unforced approach (without guide sequence). Approximately 135 kb of continuous genomic sequence were recovered for each isolate, the large genomic terminal repeats, and adjoining regions were not sequenced. MVA-A2 and MVA-CR7 were identical to the sequence of MVA (#AY603355 and corrected entry #U94848) but MVA-CR11 deviated at three positions. The affected genes in the nomenclature for vaccinia virus Copenhagen [25] are A3L, A9L and A34R; in the nomenclature for MVA [23] they are MVA114L, MVA120L, and MVA145R, respectively. The point mutations are C1915T on the coding DNA strand (or H639Y at the amino acid level) in A3L, A223G (K75E) in A9L, and G256T (D86Y) in A34R. Disregarding single-base deletions in nucleotide repeats that were considered sequencing artifacts (for example tttttat-aaaataa *versus* tttttataaaaataa in the sequence of one isolate but not present in any of the other isolates) no further sequence deviations were observed.

### 2.2. Confirmation Experiments.

**Figure 2:**
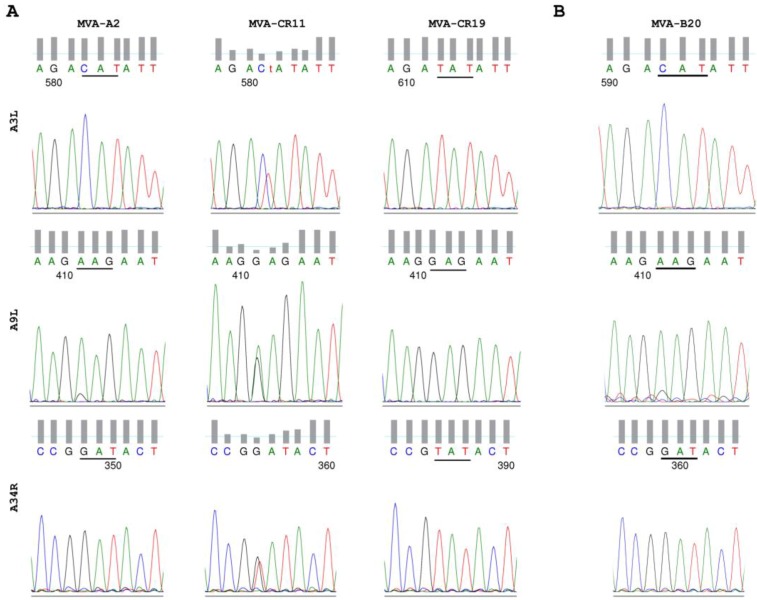
MVA-CR19 is a pure isolate of MVA-CR. (**A**) Conventional sequencing chromatograms covering the affected region of A3L, A9L, and A34R in the seed virus MVA-A2, the intermediate isolate MVA-CR11, and plaque-purified isolate MVA-CR19. (**B**) The A3L, A9L, and A34R genes remain free of mutations after repeated passages in adherent CR.pIX and mammalian R05T cells. Shown here are the sequences for isolate MVA-B20 that was obtained after 20 passages on adherent CR cells (see also Figure 5).

To confirm that the point mutations indeed indicate a transition from parental MVA to MVA-CR, we made use of a novel AccI and the loss of a BsaWI restriction enzyme site due to the G256T transversion in A34R. By digesting amplicons, obtained from MVA isolates of different passages, an increase towards the MVA-CR genotype was observed (Figure 1D). Passaging of MVA-CR was continued in suspension cultures up to passage 16. Thereafter, two consecutive rounds of plaque purification were performed, and the resulting virus therefrom (isolate MVA-CR19) again amplified in suspension cultures in chemically defined media. By AccI and BsaWI digest, the parental MVA-A2 genotype was visible only in one, and a mixed genotype only in two of a total of eleven picked clones in the first round of plaque purification (data not shown), suggesting an already advanced accumulation of the new genotype. Conventional sequencing of the three affected genes confirmed the *Next Generation Sequencing* results and revealed a mixed population of parental and MVA-CR genotypes in MVA-CR11, and pure MVA-CR genotype in MVA-CR19 (Figure 2).

### 2.3. Higher Proportion of Infectious Units in the Cell-Free Space.

**Figure 3:**
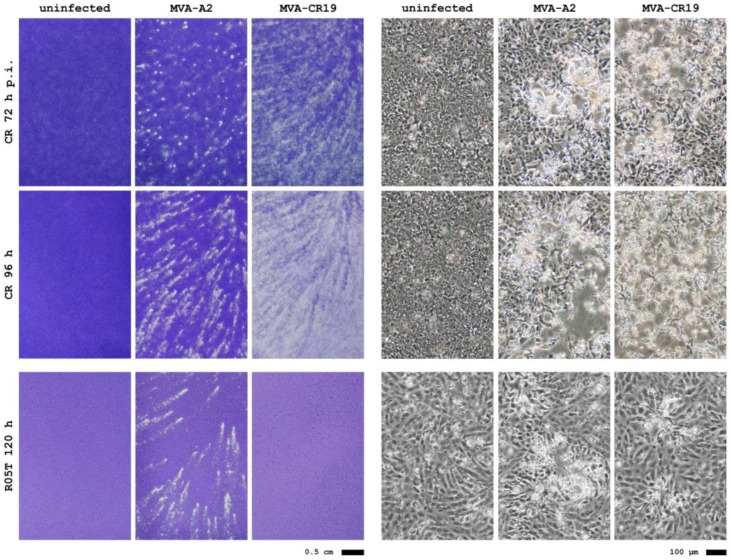
Plaque phenotypes of MVA-A2 and MVA-CR19 in CR and R05T cell monolayers stained with crystal violet (left panel) and at 40 × initial magnification of live cells (right panel). Note that MVA-CR19 causes plaques in R05T (see microscope image) but that these are too small to be visible to the unaided eye in the crystal violet-stained monolayer.

With MVA-CR19 as a pure MVA-CR isolate, properties of the new lineage were studied in greater detail. We first examined plaque phenotype of MVA-CR in adherent cultures. As shown in Figure 3, comets predominate 72 h after infection with MVA-CR19 whereas round plaques predominate at that time in CR cells infected with MVA-A2; 96 h post infection comets are clearly visible also in MVA-A2 infected cells whereas the cell layer is heavily damaged (and thus barely stainable) after infection with MVA-CR19. The assay was also performed with the R05T cell line that was obtained by immortalization of primary cells from the Egyptian Rousette. This is one [26] of very few [8] mammalian cell lines permissive for MVA. Surprisingly, an inverse relationship was observed in the two cell lines: MVA-CR19 induces greater cell damage in CR monolayers but produces only weak plaques in R05T cultures (Figure 3).

**Figure 4:**
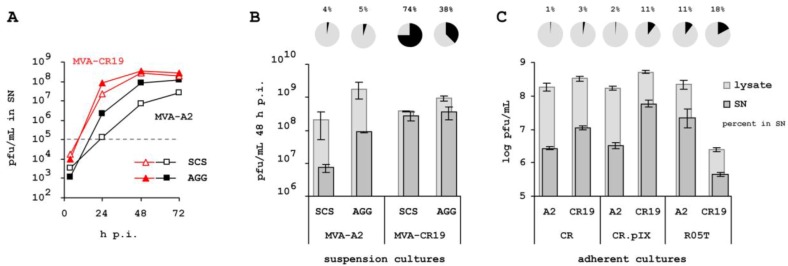
Fraction of infectious units in the supernatant of cells in suspension and adherent cultures. (**A**) Kinetic of replication of isolates MVA-A2 and MVA-CR19 in CR.pIX suspension cultures with (bold symbols) or without (open symbols) induction of cell aggregates that promote replication of various hyperattenuated poxviruses [21]. The stippled line at 10^5^ pfu/mL corresponds to input virus with an MOI of 0.05. SCS, single cell suspension; AGG, suspension culture with induced cell aggregates. Cell free supernatant was obtained by gentle centrifugation of samples for 5 min at 200 × g to avoid inducing cell lysis. The cell pellet was discarded and virus in the supernatant was subjected to three freeze/thaw cycles (−85 °C / 37 °C) to rupture the outer membrane of the EV for increased infectivity. (**B**) Distribution of infectious units in cell-free supernatant (SN) or complete lysate (supernatant and cell-associated virus) 48 h post infection. Light columns indicate pfu/mL of the complete lysate, dark columns concentration of infectious units determined in the SN. The pie charts and percentages indicate infectious units in the SN in relation to complete lysate. The curves shown in (**A**) are the average of three parallel experiments. In (**B**) standard deviation of the three repeats is shown for the 48 h-values. The samples used to obtain these data points are from the kinetic of panel (**A**). (**C**) Virus release from adherent producer cells cultivated in the presence of serum. Cultures were assayed for MVA-A2 or MVA-CR19, 48 h p.i. in infected CR or CR.pIX cells, and 144 h p.i. in R05T cells.

We next examined whether plaque phenotype in adherent cultures is also an indication of greater viral mobility within a cell suspension in chemically defined medium. We therefore infected CR.pIX suspension cultures with isolates MVA-A2 and MVA-CR19 but this time, also determined infectious units in the cell-free supernatant. We furthermore tested virus replication in a single cell culture kept in cell proliferation medium only. Normally, poxvirus replication and adequate yields are obtained only if aggregate formation is being induced by addition of a virus production medium [21]. As shown in Figure 4A, MVA-CR19, surprisingly, replicates just as efficiently in the single cell culture as in the suspension consisting of induced aggregates (red curves). As expected, titers of MVA-A2 replicating in single cell suspension are at least 10-fold below the values obtained after induction of aggregates (black curves, open symbols for replication in single-cell suspension, bold symbols for replication allowed in the presence of cell aggregates). In Figure 4B, infectious units in the supernatant are compared to the yield in a sonicated lysate of cells and supernatant, 48 h post infection. With this complete lysate as reference, a greater percentage of infectious MVA-CR19 virus is in the cell-free compartment (74.0% after replication in single cell suspension, 37.5% in cultures with induced cell aggregates) compared to MVA-A2 (3.6% and 4.9%, respectively). Although MVA-A2 replicates to higher titers than MVA-CR19 in presence of aggregates in this experiment (1.8 × 10^9^ *vs.* 9.4 × 10^8^ pfu/mL) both the absolute and relative infectious activities in the supernatant are lower.

In adherent cultures the effects are not as pronounced as in the suspension cultures. There appears to be a tendency towards higher total and extracellular yields for MVA-CR19 also in the adherent cultures, but the fraction of virus in the cell free supernatant is low for both isolates (compare Figure 4B and Figure 4C). In R05T, where MVA-CR19 replicates weakly, the relative amount of infectious virus not associated with the cell monolayer is also similar for the two lineages. From these results we would predict that the MVA-CR genotype would be difficult to rescue by passaging in adherent cultures.

### 2.4. Specificity for Suspension Process.

**Figure 5:**
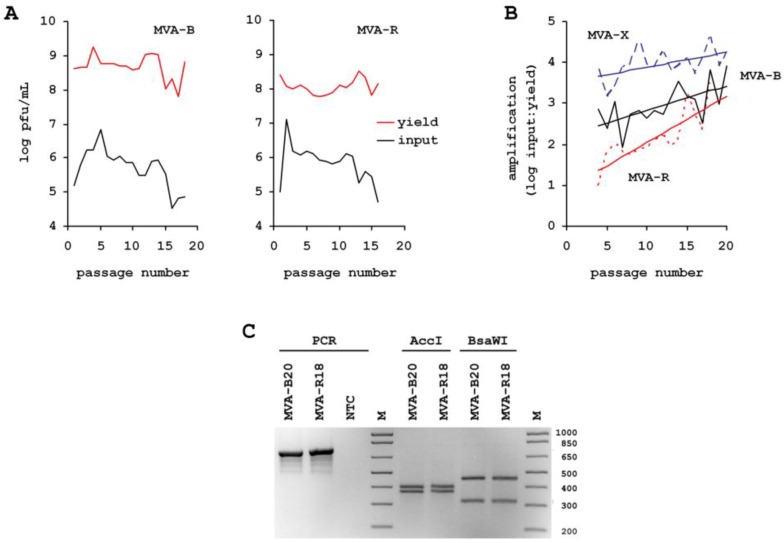
Consecutive passages of MVA on adherent serum-dependent CR.pIX cells and mammalian R05T cells. MVA-B is the lineage derived from the CR.pIX and MVA-R is the lineage derived from the R05T cells. (**A**) Sequential infection was performed without knowing the titer of the previous passage. The resulting fluctuations in input virus are shown in the lower (black) curve, the yield is shown in the upper (red) curve. Units are log_10_ of pfu/mL. Complete lysate for virus passaging was prepared from cultures with pronounced cytopathic effect, 48 h to 72 h post infection in CR.pIX cultures and 72 h to 96 h post infection in R05T cultures. Virus was released by sonication. (**B**) Dividing yield by input virus describes amplification of virus for each passage. MVA-X (broken blue curve) is the lineage that was derived from the suspension culture in chemically-defined medium shown in Figure 1. (**C**) No emergence of the D86Y A34R phenotype in MVA populations passaged on adherent cells. Compare to Figure 1D for restriction fragment length polymorphism where the D86Y genotype in A34R accumulates. Maintenance of wild type sequence in the complete genes A3L, A9L and A34R was also confirmed by conventional sequencing, with the relevant region shown for MVA-B20 in Figure 2B.

To further characterize the selective pressures driving emergence of strain MVA-CR, we also isolated successive generations of MVA from adherent CR.pIX and R05T cell lines. Passaging on the adherent CR.pIX cell line will query whether emergence of MVA-CR is influenced more by host cell characteristics rather than the chemically-defined suspension environment, passaging on R05T as a mammalian yet MVA-permissive cell line serves as an additional adherent control. The procedure mirrored the initial passaging in the CR suspension cultures: 1.5 × 10^6^ CR.pIX and 1 × 10^6^ R05T cells were seeded into T25 flasks for each passage. Infection was performed to an estimated MOI of 0.1, the actual input virus was determined by titration at later stages and is shown in the lower curve in Figure 5A. Figure 5B depicts amplification of virus, via ratio of input virus to released progeny virus for successive passages obtained from suspension CR (with data shown in Figure 1A), adherent CR.pIX or adherent R05T cultures, respectively. The efficiency of virus replication appears to increase with passage number suggesting that some adaptation of MVA occurs in all three systems. The effect appears to be greatest in the R05T cultures, which may correlate with the greater phylogenetic distance of the non-avian source to chicken, which was used for generation of MVA. However, the G256T genotype in A34R does not emerge and accumulate in any of the two adherent systems (Figure 5C), which is also confirmed for this and the two other mutations by sequencing of PCR amplicons without subcloning (data not shown).

### 2.5. Maintenance of Host-Cell Restriction of MVA-CR

**Figure 6:**
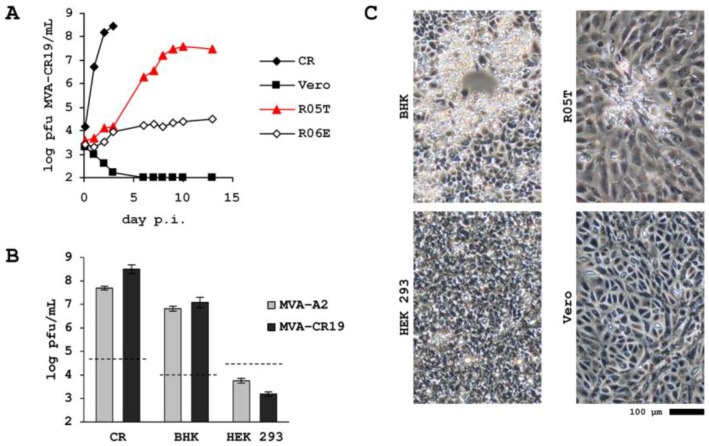
Host cell-restriction of MVA-CR isolate MVA-CR19. Cells were seeded with 5 × 10^5^ (CR), 2 × 10^5^ (R05T and HEK 293), and 1 × 10^5^ (R06E and Vero), respectively, per well of a 6-well plate and MVA was added to an MOI of 0.1. Cell lysate was prepared by freezing the plates and sonicating a thawed lysate thereof at the indicated time points. All samples were stored at −85 °C and at the end of the experiment titered together in a microfocus assay on Vero cells. (**A**) Replication kinetic of isolate MVA-CR19. (**B**) Maximum titers of parental MVA-A2 and isolate MVA-CR19 in BHK and HEK 293 cells. Infection was performed with MOI of 0.1 but variable number of cells, the stippled line depicts the amount of input virus in log_10_ pfu/mL. (**C**) Examples of cytopathic effect (or absence of cytopathic effect) in MVA-CR19-infected adherent non-avian cells, 4 days post infection for BHK and R05T, 7 days post infection for HEK 293 and Vero.

To test whether attenuation of MVA-CR can be expected to have been maintained, we infected adherent monolayers of CR, Vero, HEK 293 and R05T cell lines with MVA-A2, MVA-CR11 and MVA-CR19. The data in Figure 6 confirms expected properties of strain MVA-CR with no progeny formation in Vero, very slow replication in R06E (also a cell line from the Egyptian Rousette [26]), moderate replication in R05T, and very high productivities in CR cultures. A similar replication phenotype has been observed previously for MVA-A2 [27] in these cell lines, and is here further expanded upon in a direct comparison between MVA-A2 and MVA-CR in BHK (where MVA is expected to replicate [8]), and non-permissive HEK 293 cells.

## 3. Experimental Section

### 3.1. Cells and Viruses

Generation of the CR and CR.pIX cell lines from Muscovy duck [20], and the R05T and R06E cell lines from the Egyptian Rousette [26] has been described in detail previously. CR.pIX was derived from the CR cell line by stable transfection of an expression plasmid for the adenovirus serotype 5 protein IX (pIX). All other cell lines and modified vaccinia Ankara (MVA) were obtained from ATCC, the cell lines were authenticated by PCR [27]. Adherent cultivations were performed at 37 °C in an atmosphere enriched to 8% CO_2_. Cultivation in suspension was performed in a shaking incubator (Infors, Switzerland) with a 5 cm amplitude of the rotating platform and 180 rpm or 150 rpm for shake tubes or shake flasks, respectively, also at 37 °C in an 8% CO_2_ atmosphere. Medium for adherent cell lines was DMEM/F12 1:1 medium (Invitrogen, Carlsbad, CA) containing 5% fetal calf serum and 2 mM GlutaMAX, and, for suspension cell lines, chemically defined CD-U3 medium (PAA, Austria) supplemented to 2 mM GlutaMAX (Invitrogen) and 10 ng/mL Long-R3IGF (Sigma, St. Louis, MO). Adherent cells were infected by adding MVA directly to the medium, without any change of medium thereafter. For production of MVA in CR or CR.pIX suspension cultures [21], cells were allowed to proliferate to 4 × 10^6^ cells/mL as single cell cultures in CD-U3 medium. Thereafter, one volume of CD-VP4 virus production medium (Biochrom, Germany) was added to induce cell aggregates and the culture was inoculated with virus to a multiplicity of infection (MOI) as indicated, usually within 0.01 and 0.1 pfu/cell. Virus was recovered by sonication for 45 s with a Branson S250-D unit powering a 3.2 mm sonifier tip with 10% energy. For experiments demonstrating infectious units released by the host cells samples were centrifuged for 5 min at 200 × g. The cell pellet was discarded and virus in the SN was subjected to three freeze/thaw cycles (−85 °C / 37 °C) to rupture the outer membrane of the EV for increased infectivity.

### 3.2. Titration

The number of infectious units was determined by adding serial dilutions of a virus preparation to 80% confluent Vero monolayers. MVA cannot replicate in Vero cells so using such a substrate allows to strictly quantify only the input virus. After 48 hours, the cells were fixed with methanol and incubated with polyclonal vaccinia virus antibodies (Quartett Immunodiagnostika, Germany) at a 1:1000 dilution in phosphate-buffered saline (PBS) containing 1% fetal calf serum. Two wash steps were performed with PBS, containing 0.05% Tween 20 (Sigma), and secondary antibody to the vaccinia-specific antibody (Promega, Fitchburg, WI) was added at 1:1000, coupled to peroxidase and visualized by staining with AEC reagent (3-amino-9-ethyl-carbozole; 0.3 mg/ml in 0.1 M acetate buffer pH 5.0 containing 0.015% H_2_O_2_). Infected cells are identified by light microscopy and plaque forming units/ml are calculated by counting the number of cells positive for vaccinia virus antigens in the well infected with the maximum dilution of MVA suspension. All titrations were performed in parallel replicates (giving a total of four titration values per sample).

### 3.3. Plaque Purification

1 × 10^6^ adherent CR cells were seeded per well of a 6-well plate. After 24 h, 10^4^ total pfu of MVA was added and after a further 30 min, the culture medium was replaced with 0.8 % of low-gelling agarose (Sigma #A9045) in DMEM:F12 medium containing 5% FCS. After 72 hours approximately 2 mm-diameter agarose cores were picked from foci of cytopathic effect and transferred to a fresh adherent cell monolayer at 80% of maximum confluency in 12-well plates.

### 3.4. Plaque Phenotype

Circular plaques indicate strong cell association whereas elongated, or comet-like, plaques are formed if viruses can escape the host cell to infect more distant sites on the cell monolayer [28]. To visualize plaque phenotype, 1 × 10^6^ adherent CR or 1.5 × 10^6^ R05T cells were seeded into a T25 flask. After 24 h, 10000 (CR) or 50000 (R05T) pfu of MVA was added and the flasks kept undisturbed in the incubator for 24-96 h. The cell monolayer was fixed by addition of 0.2 volumes of 10% formaldehyde in PBS directly to the medium and stained with 0.05% crystal violet (Sigma) in water.

### 3.5. DNA and Sequencing

To isolate viral genomic DNA, 100 mL of CR cultures were infected at 2 × 10^6^ cells/mL with MVA at 0.01 MOI in a 1:1 mixture of CD-U3 and CD-VP4 media. Cells were removed by centrifugation with 200 × g, 48 h post infection and polyethylene glycol was added to a final concentration of 8% to the cleared supernatant. After incubation on ice for 30 min, the suspension was centrifuged for 60 min with 6600 × g, the translucent pellet containing viral particles was resuspended in PBS, and extraneous DNA was digested with 8 units of Turbo DNase (Invitrogen/Ambion). After inactivation of the DNase by heating to 80 °C for 10 min, DNA was isolated with the DNA blood mini preparation kit (Qiagen, Germany) according to the manufacturer’s instructions. Sequences were obtained by LGC Genomics (Berlin, Germany) with the Roche/454 GS FLX+ technology, with 50-fold coverage for each genome and assembled using an unforced (without guide sequence) algorithm.

PCR was performed with KOD HiFi DNA polymerase (TOYOBO Novagen/Millipore, Germany) with 36 cycles of 20 s 55 °C annealing, 60 s 72 °C amplification (120 s for A3L), and 20 s 94 °C denaturation. The primers for amplification and sequencing of A9L were GCAAACGCGATAAGGATACG and AAGCGGATGCAGAATAGACG, and of A34R were gCggAATCATCAACACTACCC and TAATAACAAACgCggCgTCCATggC. Sequencing of the larger A3L open reading frame was spanned with several primers: amplification was performed with GCAGAAGAACACCGCTTAGG and ATGGAAGCCGTGGTCAATAG, sequencing with the forward amplification primer, TGAGAGCTCGCATCAATC, ATCGGACTGTCGGATGTTGTG, and CTAGAATCGGTGACCAACTC. Sequences were obtained without subcloning of purified PCR products to also visualize mixtures of genotypes in the chromatograms. Purification was performed by agarose gel electrophoresis and isolation with the QIAquick Gel Extraction kit (Qiagen) according to the manufacturer’s instructions.

Mutation D86Y in A34R introduces a target site for the AccI (New England BioLabs, Ipswich, MA) restriction enzyme: from agaccg**g**atact to agaccG↓**T**ATACt. At the same time, the BsaWI (New England BioLabs) site of the wildtype is lost: from agA↓CCG**G**Atact to agaccg**t**atact. Digestion of the 772 bp A34R amplicon with AccI yields 399 and 373 bp for the parental MVA, and 399, 316 and 57 bp for MVA-CR. Conversely, digestion with BsaWI (at 60 °C) yields 452 and 320 bp for parental MVA and the uncut 772 bp for MVA-CR. The fragments were separated by 3% agarose gel electrophoresis in TAE buffer.

## 4. Discussion and Conclusions

### 4.1. Enrichment of a New Genotyp

Vectored vaccines are expected to lead to new approaches for treatment of infectious diseases and certain cancers [29]. However, often, they are also very complex products difficult to provide in sufficient amounts. Although high yields at large volumes can be achieved with suspension cultures, the nature and extent of selection pressures exerted on some vaccine strains by chemically defined production processes with suspension cultures may not be fully appreciated. In one such production process, developed specifically for MVA, and shown to produce this virus to high titers [21], we obtained a fast enrichment of a genotype characterized by three coding point mutations in the genes MVA114L (also referred to as A3L in vaccinia virus Copenhagen), MVA120L (A9L), and MVA145R (A34R). Within 16 virus passages, this genotype predominated to an extent that, in plaque purification, nine out of 11 picked isolates were positive for the D86Y mutation in MVA145R (characterized by restriction enzyme digest; data not shown). *Next Generation Sequencing* of an advanced isolate (MVA-CR11 in Figure 1) revealed only the, here described, three point mutations in 135 kb of recovered genomic sequence. We detected no additional coding or silent mutations. All of the recovered regions in MVA-CR11, as well as that of the seed virus and of an intermediate isolate (MVA-CR7) were identical to the published sequence of MVA (GenBank entries #AY603355 and corrected #U94848) again highlighting the exceptional genomic stability of MVA.

Conventional sequencing directly on PCR fragments of the affected genes revealed mixed populations of parental and MVA-CR genotypes in intermediate passages prior to plaque purification. We were unable to discover MVA-CR in early passages but detected this genotype in two independent isolates (starting with MVA-X14 and MVA-CR11) that suggests that our seed virus preparation MVA-A2 may not be genetically homogeneous. However, while certain mutations may be part of parental MVA-A2, analysis of two additional independent lineages (MVA-B and MVA-R) indicates that selection towards the MVA-CR genotype is driven by the environment (suspension media and culture process) rather than host cell type (*Cairina moschata* as source for the CR cell lines, instead of chicken used for the attenuation of MVA): after more than 18 passages of parental MVA on adherent cultures in conventional basal medium containing 5% FCS we did not observe emergence of the MVA-CR genotype, neither by restriction enzyme analysis, nor by direct sequencing of the MVA114L, MVA120L and MVA145R genes (Figure 2, Figure 5, and data not shown). The adherent cultures consisted of CR.pIX cells directly related to the suspension CR cell line and of R05T cells, a fruit bat cell line previously shown to be fully permissive for MVA.

The three affected genes, separated by 6 kb (A3L to A9L), and 21 kb (A9L to A34R) on the genomic DNA, are expressed late in the infectious cycle and encode structural proteins that appear to occupy different layers in the complex events leading to infectious viral particles (for example reviewed by [30,31]). Particle formation starts in cytoplasmatic sites called virus factories. Therein, host derived lipids and viral proteins assemble into crescent shapes, and from there, into the spherical and non-infectious immature virions (abbreviated as IVs or IMVs). The A3 and A9 proteins both appear to be involved at early stages of maturation of the IV. A3 (or P4b) is one of three major core proteins, and processed by the I7L-encoded viral protease [32]. Probably in cooperation with the highly abundant major core protein P4a precursor encoded by A10L (MVA121L), it may play an important role in correct core wall formation, and DNA condensation, preparing the IV for morphogenesis towards the stable and infectious mature virus particle (MV or IMV) [33,34]. Thereafter, the A9 protein, most likely as a component of the MV membrane in a postcrescent stage, appears to contribute to establishing a connection between core and membrane in the final steps of transition to the MV [35].

The third affected protein (A34) appears to be involved very late in the infectious cycle. First, only a fraction of the MVs are transported along microtubules towards the trans-Golgi network where they are enveloped by cell-derived vesicles. The resulting intracellular enveloped virion, thus, gains a double membrane and the core is now wrapped by three membranes. Still driven by microtubules, the MV reaches the cell periphery where only the outer of the vesicles-derived viral membranes fuses with the plasma membrane. The resulting cell-associated doubly-enveloped virus can remain attached to the host cell, or can be propelled along actin tails to infect new cells in the vicinity of the producer cell; it can also detach, forming the enveloped extracellular virus (EV, previously abbreviated as EEV), to spread the infection to more distant sites. Compared to the MV, the EV has an additional membrane that helps to protect the infectious kernel from the adaptive immune system. However, the additional membrane of the EV is not equipped to mediate fusion with the target cell and must be disrupted to expose the MV, the actual infectious unit. Studies with A34R (MVA145R) deletion mutants demonstrated that this factor is an important determinate for infectious activity in the extracellular space and for spread of vaccinia virus [36], a function that the A34 protein probably mediates by destabilizing the EV outer membrane. Furthermore, A34, together with A33R and B5R gene products [37,38,39], also appears to modulate the rate at which the cell-associated virus detaches from the host cell. Quantification of intracellular, cell-associated and extracellular infectious units, data that we have not attempted to obtain in this study, may help to delineate whether the mechanism that contributes to extracellular titers of MVA-CR in suspension cultures is connected to changes in outer membrane stability or EV detachment.

### 4.2. Extracellular Infectious Units

The discovered targets of the mutations appear consistent with our observation that infectious activity in the suspension obtained with the MVA-CR genotype appears to be increased, and with our hypothesis that MVA-CR may be released more easily into the extracellular space than parental MVA-A2. As shown in Figure 4, 75% of MVA-CR can be recovered from a cell-free suspension whereas only 5% of MVA-A2 was observed in this compartment. Greatest accumulation of infectious units in the supernatant was observed in an infected culture, maintained only in cell proliferation medium. We have shown previously [20,21] that MVA replication in suspension cultures is inefficient in media that support single-cell suspensions and that yields are significantly increased by addition of a medium that induces cell aggregate formation. This requirement is a property of MVA; influenza A virus was shown to replicate in conventional CR.pIX cell suspension cultures [40]. In the biphasic process where aggregates are induced at the time of infection, the proportion of extracellular MVA-CR is reduced to 38% but still greater than the 5% measured for MVA-A2. That more MVA-CR19 virus appears to be trapped within (or attached to) host cells in presence of virus production medium is consistent with our intention of facilitating cell-to-cell spread of the virus. In adherent cultures (shown in Figure 4), infectious units of MVA-A2 and MVA-CR19 were found predominantly in the full lysate and not in the supernatant. In cell cultures, any shielding properties of the EV (reviewed in [41,42]) such as interference with complement cascades of the immune system, are not expected to convey an advantage. In such an environment, the outer membrane of the EV may simply interfere with infectivity of MVA, and one reason for increased extracellular infectivity of MVA-CR in suspension cultures could be due to decreased stability of this component. Without culture agitation, and in presence of virion-stabilizing fetal calf serum, the outer membrane of MVA-CR may be retained for greater time intervals, leveling this potential advantage of MVA-CR. Furthermore, concentration of divalent cations are known to promote cell adherence and are therefore elevated in media designed for anchorage-dependent cultures. Increased concentration of Mg^2+^ and Ca^2+^ probably also strengthens the association of virus envelopes to plasma membranes, again leveling any advantages that we observed for MVA-CR in chemically defined suspension medium.

Provided that all safety considerations are fulfilled, a strain of MVA that yields greater infectious activity in the culture supernatant provides two important advantages. Currently, production requires addition of one volume of medium formulated to facilitate virus replication but interfering with cell proliferation. Although the process is robust, if cell proliferation and virus production can be performed in a single medium vaccine manufacturing could be simplified. The effect could be that the process is yet more transportable near to geographic regions where some vaccines are most urgently required, and local regulatory approval processes are preferred. Furthermore, during peak titers the cytopathic effect in the host cell is still low and the cells remain largely intact. If a greater proportion of MVA is released, the cells can be removed and fewer host cell components will copurify into the final preparation. For any vaccine derived from a continuous cell line, residual DNA should be present at a level of 10 ng per dose, or less [19]. If only culture supernatant, and not a complete lysate, needs to be purified, such levels should be obtained more easily. Because downstream processing contributes significantly to the costs of injectable vaccines [43] a strain with simplified purification requirements may help to reduce economic pressures on MVA-based vaccines.

### 4.3. Attenuation on R05T

Although MVA-CR spreads more easily and produces larger foci in the cognate duck cell line, attenuation of this virus for mammalian cells may actually be reduced. As shown in Figure 6, while MVA-CR replicates to expected titers in CR, the Vero cell line remains non-permissive. Interestingly, the R05T cell line, which normally is fully permissive, does not support MVA-CR19 replication as efficiently as MVA-A2. Furthermore, plaques caused by MVA-CR19 are far less pronounced than plaques caused by MVA-A2. While improved detachment of MVA-CR may be sufficient to explain the increase of infectious units in the supernatant, especially in duck suspension cell lines, the eclipse period of MVA-CR19 in R05T appears to be longer than the eclipse of MVA-A2, suggesting that lower replication rates and smaller foci could also be due to less efficient entry or uncoating of virus. It is tempting to speculate that due to their proximity to the genomic nucleoid, the mutated A3 and A9 proteins affect this property, but there appear to be currently no data to support such a model. At this time, the mutation in the A34 protein appears to be a highly probable candidate for the observed attenuation effects. There are reports consistent with the impact of this factor, also on EV membrane stability at the time of infection [44], and since MV and EV may gain entry by different mechanism [41,42] the relative abundance of these virion forms may explain differences in infectious activity depending on the type of host cell. For example, attachment of the MV appears to be mediated by interaction between cellular glycosaminoglycans and the viral A2L (MVA138L), D8L (MVA105L), and H3L (MVA093L) gene products [31]. This attachment has been shown to be influenced by the cell type [45] and, perhaps, the fruit bat cell line is at a critical threshold of appropriate plasma membrane decoration with glycosaminoglycans. In this context it is also possible that less fluid actin rearrangement, or less rapid pH changes in the R05T cell line, may have a role in contributing to decreased susceptibility to MVA-CR19 compared to MVA-A2. Regarding other candidate factors, neither the F13L gene (MVA043L), also shown to impact vaccinia virus detachment [46,47] and attenuation [48], appears to be mutated in MVA-CR, nor proteins implicated as components of the entry fusion complex of vaccinia virus [31], including the above mentioned A27L, D8L, and H3L genes. With respect to additional mammalian cell lines and as has been demonstrated for MVA previously [8], MVA (and MVA-CR19) replicates in BHK, but there appears to be no amplification beyond input virus in HEK 293 (Figure 6).

In summary, we have isolated a strain of MVA that appears to be adapted to replication in avian producer cells cultivated in suspension in chemically defined medium. Within the 135 kb of genomic sequence that we recovered of MVA-CR11, an isolate with the same phenotype as the plaque-purified isolate MVA-CR19, only three proteins were found each to carry a single amino acid exchange. We currently do not know whether all three factors need to cooperate to produce the observed effects or whether a single gain of function mutation in any one or two factors is sufficient. However, it is surprising that all three of the observed mutations target a different component of the complex viral particles, the core, and the different membranes of the MV and EV. With respect to safety and application as vector, host cell-restriction of MVA-CR appears not to be affected in Vero and HEK 293 cells. Although attenuation has yet to be confirmed, the observed host-cell restriction is a very promising result, suggesting that such a property can be expected also *in vivo*. Supply of an injectable vaccine preparation may be facilitated with this strain, as production in single cell suspension is less complex compared to the current protocol that requires cell aggregate induction. Furthermore, MVA-CR has a tendency to accumulate in the cell-free volume. Purification of live virus out of a cell-free suspension may provide higher yields than a process initiated with a complete lysate that contains the full burden of unwanted host cell-derived components.
